# Brain structural alterations in young girls with Rett syndrome: A voxel-based morphometry and tract-based spatial statistics study

**DOI:** 10.3389/fninf.2022.962197

**Published:** 2022-09-08

**Authors:** Dongyun Li, Lianni Mei, Huiping Li, Chunchun Hu, Bingrui Zhou, Kaifeng Zhang, Zhongwei Qiao, Xiu Xu, Qiong Xu

**Affiliations:** ^1^Department of Child Health Care, Children's Hospital of Fudan University, Shanghai, China; ^2^Department of Radiology, Children's Hospital of Fudan University, Shanghai, China

**Keywords:** voxel-based morphometry, tract-based spatial statistics, Rett, MECP2, children

## Abstract

Rett syndrome (RTT) is a neurodevelopmental disorder caused by loss-of-function variants in the *MECP2* gene, currently with no cure. Neuroimaging is an important tool for obtaining non-invasive structural and functional information about the *in vivo* brain. Multiple approaches to magnetic resonance imaging (MRI) scans have been utilized effectively in RTT patients to understand the possible pathological basis. This study combined developmental evaluations with clinical severity, T1-weighted imaging, and diffusion tensor imaging, aiming to explore the structural alterations in cohorts of young girls with RTT, idiopathic autism spectrum disorder (ASD), or typical development. Voxel-based morphometry (VBM) was used to determine the voxel-wised volumetric characteristics of gray matter, while tract-based spatial statistics (SPSS) was used to obtain voxel-wised properties of white matter. Finally, a correlation analysis between the brain structural alterations and the clinical evaluations was performed. In the RTT group, VBM revealed decreased gray matter volume in the insula, frontal cortex, calcarine, and limbic/paralimbic regions; TBSS demonstrated decreased fractional anisotropy (FA) and increased mean diffusivity (MD) mainly in the corpus callosum and other projection and association fibers such as superior longitudinal fasciculus and corona radiata. The social impairment quotient and clinical severity were associated with these morphometric alterations. This monogenic study with an early stage of RTT may provide some valuable guidance for understanding the disease pathogenesis. At the same time, the pediatric-adjusted analytic pipelines for VBM and TBSS were introduced for significant improvement over classical approaches for MRI scans in children.

## Introduction

Rett syndrome (RTT; OMIM #312750) is a neurodevelopmental disorder predominantly affecting females that is caused by loss-of-function variants in the X-linked gene encoding methyl-CpG-binding protein 2 (MECP2; Amir et al., 1999; Ip et al., 2018). RTT is characterized by relatively normal development during the first 6–18 months of life followed by stagnation regression of speech and social skills, cognitive disabilities, gait dysfunctions, and stereotypic hand movements (Neul et al., 2010; Katz et al., 2016). These behavioral impairments have been associated with global atrophy of the brain and, at the cellular level, the reduced cell body of the neuron as well as the decreased dendritic length (Reiss et al., 1993; Armstrong et al., 1995; Bauman et al., 1995; Subramaniam et al., 1997).

As a non-invasive tool, magnetic resonance imaging (MRI) has been applied to patients with RTT for localizing and quantifying anatomical differences in the past decade (Casanova et al., 1991; Reiss et al., 1993; Subramaniam et al., 1997). Some recent morphometric and volumetric studies with relatively novel methods applying voxel-based or surface-based measurements have identified selective gray matter reductions in specific areas (e.g., frontal and temporal lobes, caudate nucleus, thalamus, and cerebellum) as well as decreased FA values in the main fibers comprising the limbic system (e.g., corpus callosum, internal capsule, and frontal white matter; Carter et al., 2008; Mahmood et al., 2010; Shiohama et al., 2019). However, these previous studies used low-resolution imaging, subjects from a wide age range, or a very small sample size.

Using modern neuroimaging techniques, voxel-based morphometry (VBM) and tract-based spatial statistics (TBSS) make it possible to perform automated and unbiased whole brain analysis to determine the voxel-wised alterations of neural structures (Gunbey et al., 2017; Papma et al., 2017). It is worth mentioning that VBM was based on scans from high-resolution T1-weighted sequences and TBSS could derive data from DTI sequences even with fewer directions. Both of these two sequences are broadly available in routine clinical protocols. Unfortunately, the tissue probabilistic maps or brain templates embedded in the classical procedures of VBM and TBSS analyses were generated from scans of adults' brains. Thus, pediatric population-specific pipelines are highly recommended to solve the methodological limitations of traditional VBM and TBSS procedures when applied to datasets from children.

In this study, we employed VBM and TBSS to obtain the voxel-wised volumetric characteristics of gray matter as well as voxel-wised properties of white matter in cohorts of young girls with RTT, idiopathic autism spectrum disorder (ASD), and typical development. In this study, we primarily aimed to investigate the morphometric abnormalities and the correlations with some clinical features in young children with RTT and secondarily tried to suggest the pediatric-adjusted analytic pipelines for VBM and TBSS.

## Materials and methods

### Subjects

A total of 28 girls diagnosed with Rett syndrome based on the up-to-date diagnostic criteria (Neul et al., 2010) and confirmed with *MECP2* variants were consecutively recruited between February 2017 and November 2021 from the Division of Child Health Care, Children's Hospital of Fudan University. Following quality control, the imaging data of three RTT girls were excluded. Age-matched 30 typically developing (TD) girls were recruited as TD controls, and three of them were excluded after an image quality check. They had an MRI scan because they had their first episode of febrile convulsion, paroxysmal dizziness, or headache, but otherwise with normal development. Age-matched 32 ASD girls without known genetic etiology were recruited as idiopathic ASD controls, and four of them were excluded after an image quality check. ASD was diagnosed based on the *Diagnostic and Statistical Manual of Mental Disorders, 5th Revision* (*DSM-5*; Battle, 2013) and the *Autism Diagnostic Observation Schedule-The Second Edition* (*ADOS-2*; Gotham et al., 2007). Children with cerebral palsy, or other neurologic or degenerative diseases, or with recognizable lesions or abnormalities on scans, were excluded. The Griffiths Development Scales-Chinese (GDS-C), which consists of five developmental domains including gross motor, fine motor, social, language, and performance, was conducted to assess developmental and cognitive levels (Luiz et al., 2004; Tso et al., 2018). The Rett Syndrome Severity Scale (RSSS; Hoffbuhr et al., 2001; Kaufmann et al., 2012), which consists of seven domains including seizures, respiratory abnormalities, scoliosis, walking ability, hand use, speech, and sleep, was applied to assess the clinical severity of RTT.

This study was approved by the Ethics Committee of the Children's Hospital of Fudan University, and all of the procedures were in accordance with the Declaration of Helsinki.

### MRI data acquisition

All MRI images were scanned on a GE 3.0 Tesla Discovery MR750 system (GE Medical Systems, Milwaukee, WI) with an eight-channel head coil at the Radiology Department after parental consent. Approximately 1 h before the MRI scan, children were routinely sedated in the “Sedation Center” under the supervision of an anesthesiologist or licensed pediatrician. Chloral hydrate was given at a dose of 50 mg/kg orally, and vital signs were monitored during the scan. The high-resolution T1-weighted BRAVO (BRA in Volume imaging) sequence was performed, and the spin-echo, echo planar sequence was used to obtain the diffusion tensor imaging (DTI). T1-weighted sequence parameters: TR (repetition time) = 8.2 ms; TE (echo time) = 3.2 ms; voxel size = 1 × 1 × 1 mm^3^; matrix = 256 × 256; flip angle = 12°; gap = 0; FOV = 256 mm. DTI sequence parameters: 15 directions; b = 1,000 s/mm^2^; TR = 4,600 ms; TE = 87.4 ms; voxel size = 2^*^2^*^4 mm^3^; matrix = 128 × 128; flip angel = 90°; gap = 0; FOV = 240 mm; 15 b-factors 0 and 1,000 s/mm^2^.

Each scan was visually inspected and images containing obvious artifacts or with a low image quality rating (IQR; <0.8) were excluded. All of the original DICOM imaging files were first converted to NifTi format using the dcm2niigui toolbox within the MRIcron package (www.mricro.com/mricron).

### Voxel-based morphometry (VBM) analyses

**Figure 1:**
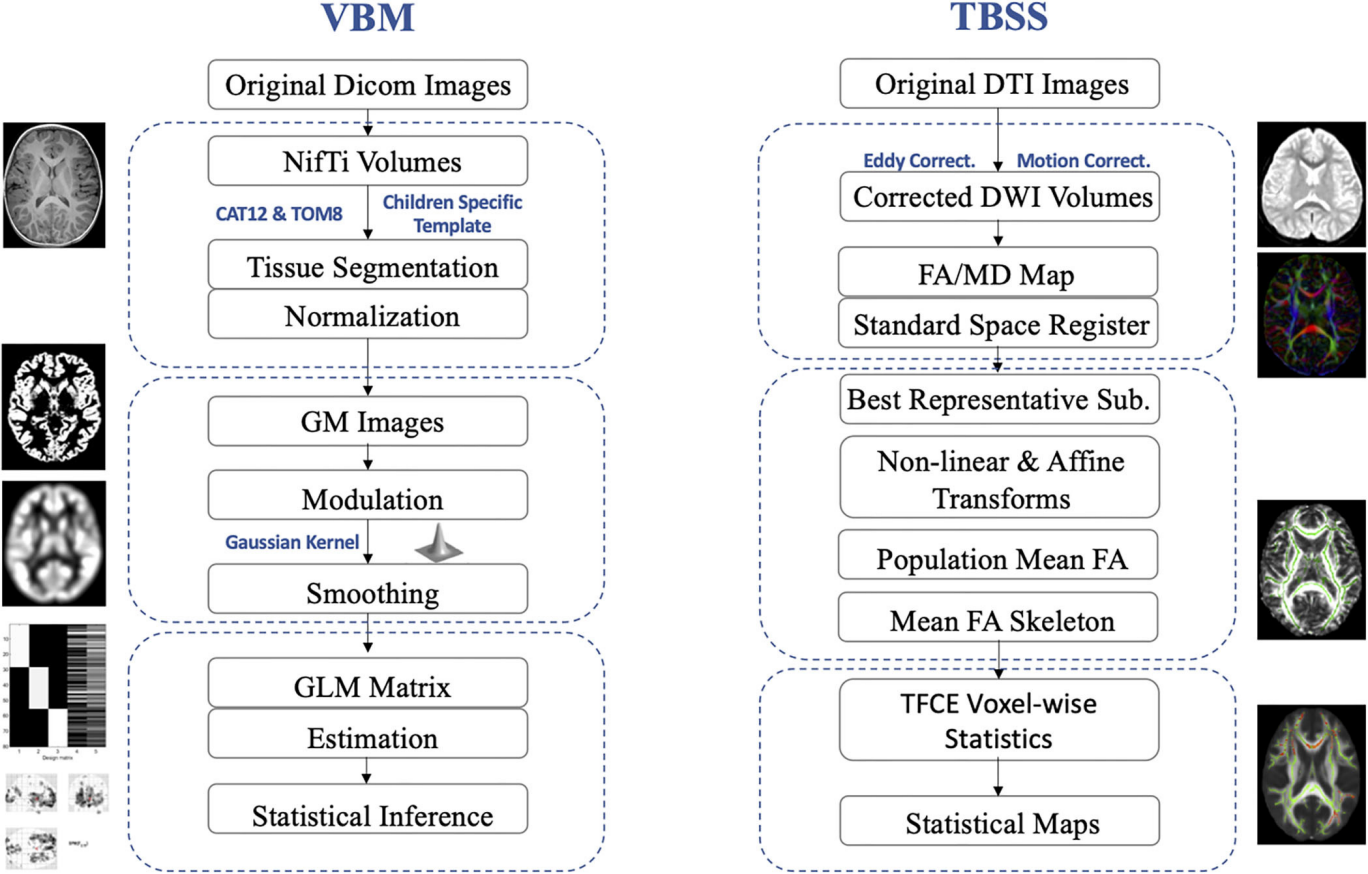
Pediatric-adjusted pipelines for VBM and TBSS. VBM, voxel-based morphometry; TBSS, tract-based spatial statistics; GM, gray matter; DTI, diffusion tensor imaging; DWI, diffusion-weighted image; FA, fractional anisotropy; MD, mean diffusivity; GLM, general linear model; TFCE, threshold-free cluster enhancement.

VBM analyses were conducted using the CAT12 toolbox (http://dbm.neuro.uni-jena.de/cat/; Gaser and Dahnke, 2016) implemented in the Statistical Parametric Mapping software package (SPM12; http://www.fil.ion.ucl.ac.uk/spm/software/spm12/; Ashburner and Friston, 2005) on the platform of Matlab (version R2019b, Mathworks Inc., USA). Population-specific tissue probability maps were created using the Template-O-Matic toolbox (Wilke et al., 2008). A fast diffeomorphic registration algorithm (Diffeomorphic Anatomical Registration using Exponentiated Lie algebra, DARTEL) was then applied to these tissue probability maps to produce the templates for subsequent normalization and segmentation. Gray matter segmented images, which aligned to the population-specific template, were then submitted to the Check Sample Homogeneity through the embedded function in CAT-12. Then, the segmented gray matter images were smoothed with a 6 mm full-width at half maximum (FWHM) Gaussian kernel. At the end of this preprocessing, the modulated, normalized, and smoothed gray matter imaging data were obtained for further statistical analyses (Figure 1 for pipeline demonstration).

### Tract-based spatial statistics (TBSS) analyses

DTI data were analyzed using the FSL software (Functional MRI of the Brain Software Library, FMRIB; Jenkinson et al., 2012). Then, the preprocessing was conducted with corrections for eddy current-induced artifacts and head motions, removing non-brain tissues, and estimating the diffusion tensor using the FDT toolbox embedded in FSL. The maps for fractional anisotropy (FA) and mean diffusivity (MD) were then calculated. Then, TBSS was applied to assess group-level differences in the DTI parameters (FA and MD; Smith et al., 2007). To adjust for pediatric imaging, a study-specific image registration procedure was applied. Then each subject's FA image was non-linearly aligned with every other's images. The FA image with the smallest average warp score obtained from registration with every other's images was identified as the “best representative” template as a target for non-linear registration. This “best representative” target image was then affine-aligned into the Montreal Neurological Institute's (MNI) 152 standard space. Every FA map was transformed non-linearly to the target image with the affine transform from that target to the MNI152 space. Then, the mean FA images and mean FA skeletons were computed as the input for TBSS. Statistical analyses were conducted based on projecting all subject's FA and MD values, respectively, onto the mean FA skeleton and submission of the 4D-projected FA and MD data (Figure 1 for pipeline demonstration).

### Statistical analyses

The statistical package SPSS 20.0 was applied for the demographic and clinical data analyses. The statistical significance level was set at *p* < 0.05. Total intracranial volume (TIV) and age were included as nuisance covariates.

For VBM, a general linear model (GLM, specified second level) was applied on the voxel-wised level. For TBSS, non-parametric permutation tests were applied for group comparisons of both FA and MD values. The statistical maps were processed at the cluster level with threshold-free cluster enhancement (TFCE). Then, an analysis of covariance (ANCOVA) was conducted among the three groups, and significant clusters were further picked up as masks for *post-hoc* tests. *Post-hoc t*-tests were then used to compare each two groups (RTT vs. ASD; RTT vs. TD; ASD vs. TD). The significant results of ANCOVA were included as an explicit mask for the *post-hoc* between-group tests. Family-wise error (FWE) corrected at *p* < 0.05 was set as a significant level with a minimum cluster size of 100 voxels.

To further investigate the correlations between clinical features and morphometric abnormalities, we first defined regions of interest (ROIs) with significant VBM and TBSS results between the RTT group and ASD controls. Then, the specific gray matter volumes of each subject were extracted for the above gray matter ROIs using ImCalc toolbox embedded SPM12 and “fslmaths” tools from FSL. FA and MD values of each subject were extracted based on the TBSS ROIs separately applying “cluster” and “fslmaths” tools embedded in FSL. Linear regression analyses were then performed for multiple corrections with a statistically significant level at *p* < 0.05.

## Results

### Participant characteristics

All the children from three groups are young girls with no significant differences in age (for RTT group, mean ± SD: 3.5 ± 1.25 years; for ASD group, mean ± SD: 3.6 ± 1.45 years; and for TD group, mean ± SD: 3.8 ± 1.22 years) between groups (all *p*-values > 0.05). Compared to the ASD group, girls with RTT exhibited significantly worse performance in all of the developmental domains of the Griffith Development Scales (all 5 domains: *p* < 0.0001). The RSSS clinical severity score was 5.76 ± 1.17 (mean ± SD) in the RTT group. No group differences were found in all of the imaging quality control parameters, including the six absolute head motion parameters and IQRs (for RTT group, mean ± SD: 0.886 ± 0.03; for ASD group, mean ± SD: 0.884± 0.02; and for TD group, mean ± SD: 0.883 ± 0.02; *p* > 0.05). Detailed demographic information and clinical characteristics are presented in Table 1.

**Table 1:** Summary of clinical characteristics of study subjects.

	**RTT group**	**ASD group**	**TD group**	***P*-value**
**T1w-VBM**				
Number of subjects	25	28	27	
Age (M ± SD)	3.5 ± 1.25	3.6 ± 1.45	3.8 ± 1.22	0.658^a*^
**DTI-TBSS**				
Number of subjects	22	23	23	
Age (M ± SD)	3.4 ± 0.81	3.5 ± 1.06	3.8 ± 1.18	0.359^a*^
**Griffith scale**				
Gross motor	32.32 ± 9.84	69.32 ± 16.00	NA	9.994^c****^
Social	15.48 ± 7.76	56.60 ± 17.65	NA	10.72^c****^
Language	14.96 ± 7.13	46.00 ± 17.74	NA	8.173^c****^
Fine motor	11.60 ± 6.93	55.71 ± 17.54	NA	11.77^c****^
Performance	10.80 ± 5.24	54.89 ± 17.86	NA	11.88^c****^
**RTT-RSSS**	5.76 ± 1.17	NA	NA	
**IQR**	0.886 ± 0.03	0.884 ± 0.02	0.883 ± 0.02	0.687^a*^
**TIV**	1200.80 ± 126.99	1437.36 ± 174.86	1432.67 ± 142.56	20.83^b****^
**GMV**	626.72 ± 56.98	795.14 ± 70.09	785.93 ± 55.30	61.43^b****^
**WMV**	289.60 ± 37.91	393.57 ± 61.47	400.11 ± 54.83	35.52^b****^
**FA**	0.137 ± 0.006	0.147 ± 0.008	0.149 ± 0.009	15.29^b****^
**MD**	0.896 ± 0.048E-03	0.867 ± 0.028E-03	0.863 ± 0.038E-03	5.17^b**^

^a^One-way ANOVA.

^b^ANCOVA.

^c^Unpaired Student's t-test (two-tailed).

*p < 0.05, **p < 0.01, ****p < 0.0001.

T1w, T1-weighted; VBM, voxel-based morphometry; DTI, diffusion tensor imaging; TBSS, tract-based spatial statistics; IQR, image quality rating; TIV, total intracranial volume; GMV, gray matter volume; WMV, white matter volume; FA, fractional anisotropy; MD, mean diffusivity; NA, not applicable.

### VBM analyses

**Figure 2:**
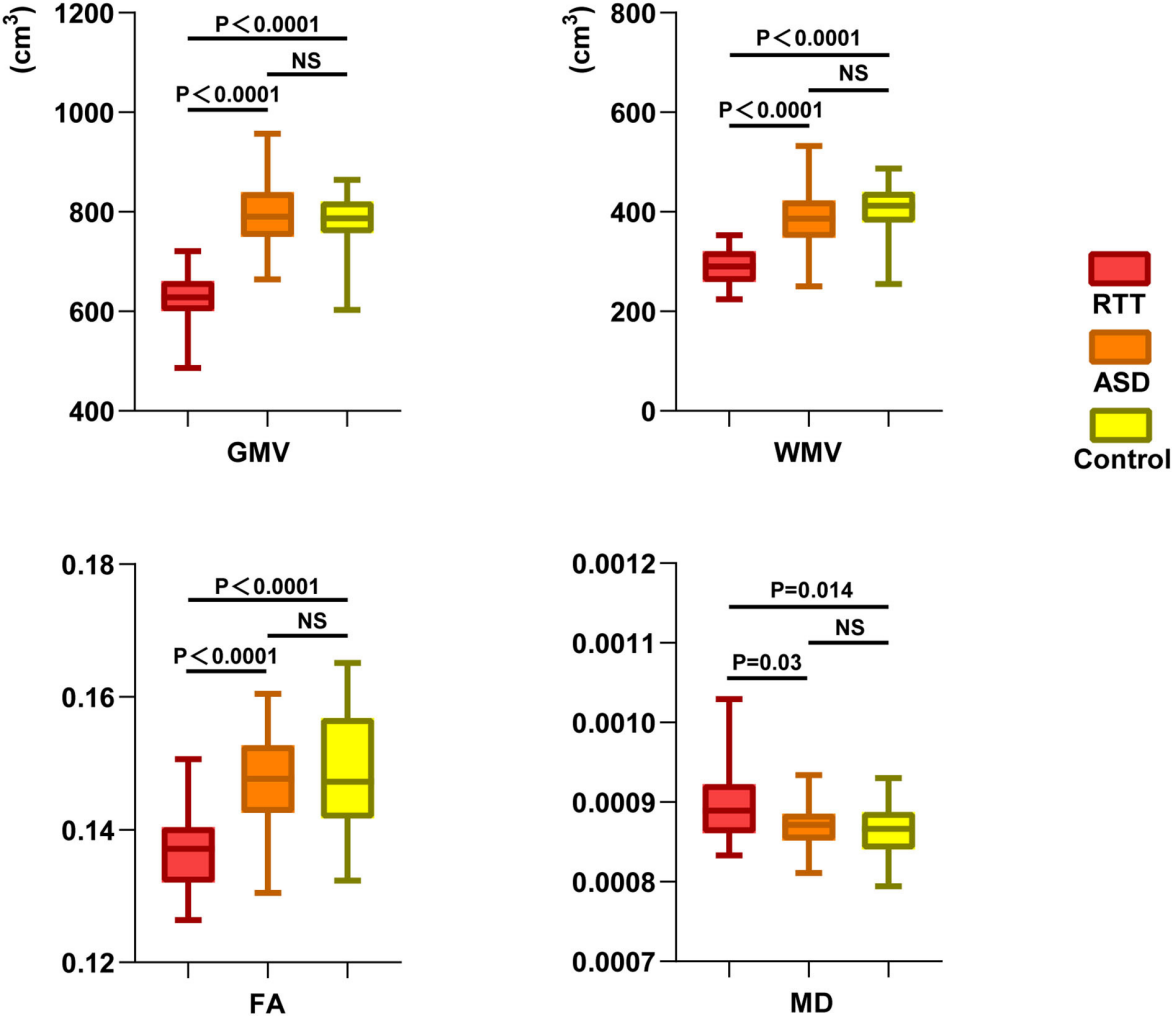
Global-leveled ANCOVA analysis showed a significant difference in gray and white matter parameters among the three groups. RTT group exhibited significantly decreased GMV, WMV, FA, and increased MD values compared to ASD and TD controls. GMV, gray matter volume; WMV, white matter volume; FA, fractional anisotropy; MD, mean diffusivity; NS, not significant.

At the global level, morphometry analysis of the whole brain showed that the RTT group exhibited a significant reduction in TIV, GMV, and WMV compared to ASD and TD controls (Table 1 and Figure 2).

VBM analysis and group comparisons were conducted between RTT group (*n* = 25), ASD controls (*n* = 28), and TD controls (*n* = 25). Through the voxel-wise ANCOVA analysis of brain volume, we found significant differences among the three groups with 8 clusters mainly in the regions of the insula, superior frontal cortex, middle frontal cortex, inferior frontal cortex, orbitofrontal cortex, calcarine, precuneus, rectus, cuneus, putamen, and hippocampus (*p* < 0.05, FWE corrected).

**Figure 3:**
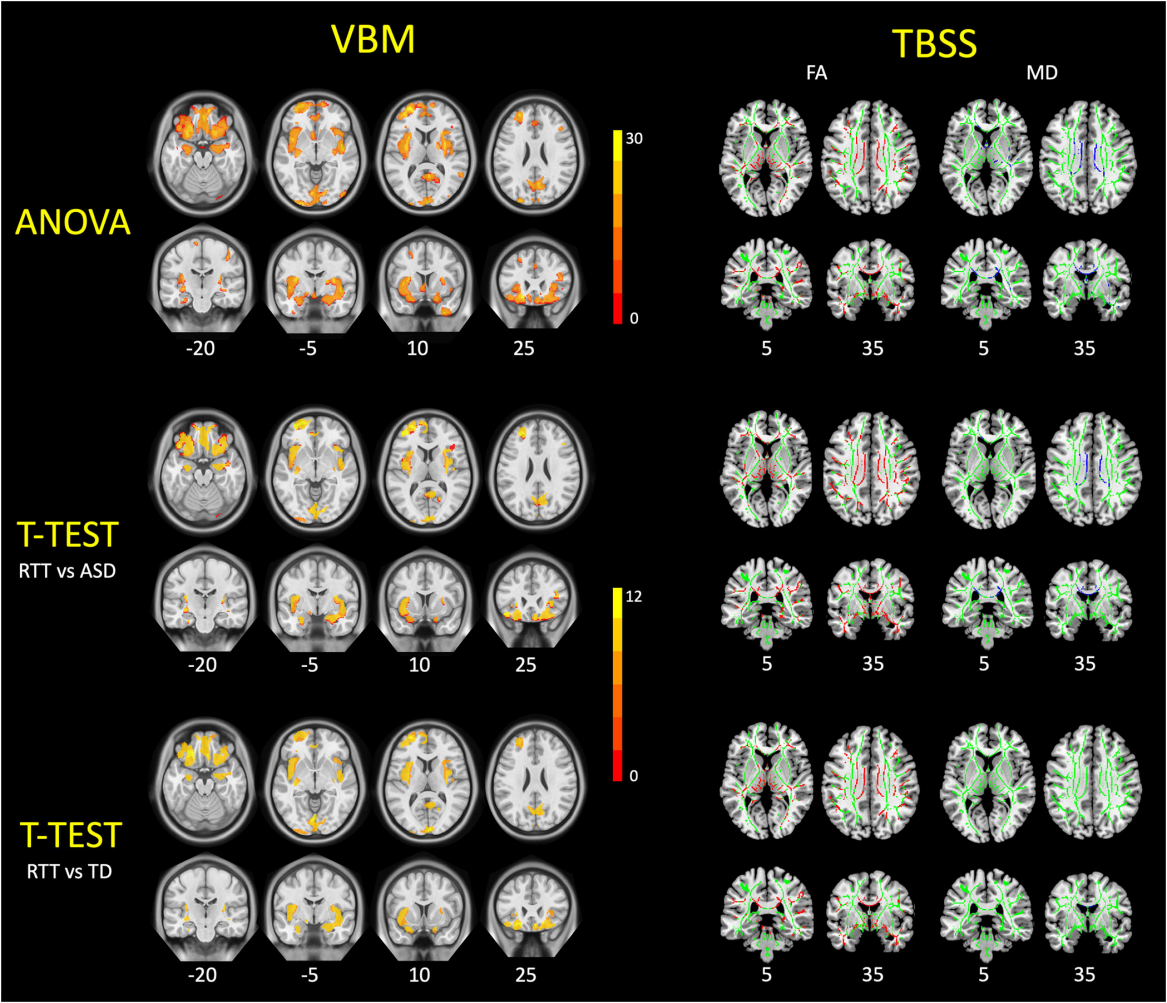
VBM and TBSS findings in young girls with RTT compared to ASD and TD controls. These figures demonstrated the ANCOVA and *post-hoc* statistical results of VBM analysis **(left)** and TBSS analysis **(right)** among three groups. For VBM results, the RTT group showed significantly decreased gray matter volumes (red-yellow) compared with controls (*p* < 0.05, FWE corrected). For TBSS results, TDD group showed significantly decreased FA values (red) and increased MD values (blue) compared with controls (*p* < 0.05, FWE corrected). TBSS results are overlaid on the mean FA skeleton (green).

In the *post-hoc* analysis, the RTT group showed significantly decreased gray matter volume compared to TD controls in four clusters (Table 2). The number of voxels in the 4 clusters was 621, 16,836, 3,818, and 1,057, respectively. The peak MNI regions were left hippocampus with coordinate (−17, −10.5, −22.5), left middle frontal cortex (−33, 49.5, −12), left calcarine (3, −99, 9), and right precuneus (−1.5, −81, 37.5), respectively. The key regions involved were the bilateral insula, calcarine, cuneus, lingual, rectus, hippocampus, and putamen, as well as the left superior frontal cortex, orbitofrontal cortex, and inferior frontal cortex (*p* < 0.05, FWE corrected; Figure 3). The comparison between RTT and ASD controls also showed significantly decreased gray matter volumes in 4 clusters (Table 2). The number of voxels in the four clusters was 543, 15,031, 2,483, and 1,697, respectively. The peak MNI regions were left para-hippocampus with coordinate (−27, −15, −27), left middle frontal cortex (−33, 49.5, −12), right lingual (12, −82.5, −7.5), and right precuneus (−10.5, −63, 27), respectively. The key regions involved were similar to the RTT-TD comparison, including the bilateral insula, calcarine, cuneus, lingual, rectus, orbitofrontal cortex, hippocampus, and putamen, as well as the left superior frontal cortex (*p* < 0.05, FWE corrected; Figure 3). However, no significant difference was found between the ASD and TD group comparisons.

**Table 2:** Decreased gray matter volumes in comparisons between RTT group and controls for VBM analysis.

**Cluster**	**1**		**2**		**3**		**4**	
**RTT** **<** **TD**
Number of voxels	621		16,836		3,818		1,057	
Peak MNI coordinate	−27, −10.5, −22.5		−33, 49.5, −12		3, −99, 9		−1.5, −81, 37.5	
Peak MNI coordinate region	Hippocampus_L	343	Frontal_Mid_L	1531	Calcarine_L 1056		Precuneus_R	717
Peak intensity	10.2694		11.8957		12.2698		4.8573	
Other involved regions	ParaHippocampus_L	140	Insula_L	2,679	Calcarine_R	583	Precuneus_L	340
(with Top10 voxelsize >100)	Putamen_L	138	Frontal_Sup_L	2,034	Lingual_R	530		
			Insula_R	1,580	Cuneus_L	529		
			OFCpost_L	1,017	Cuneus_R	352		
			Rectus_R	697	Lingual_L	311		
			Rectus_L	615	Occipital_Mid_L	278		
			Frontal_Inf_L	575	Occipital_Inf_L	118		
			Putamen_R	468				
			Hippocampus_R	404				
			ParaHippocampus_R	303				
			……					
**RTT** **<** **ASD**
Number of voxels	543		15,031		2,483		1,697	
Peak MNI coordinate	−27, −15, −27		−33, 49.5, −12		12, −82.5, −7.5		−10.5, −63, 27	
Peak MNI coordinate region	ParaHippocampus_L	183	Frontal_Mid_L	1354	Lingual_R	551	Precuneus_R	632
Peak intensity	8.6294		11.6149		10.8569		9.6658	
Other involved regions	Hippocampus_L	252	Insula_L	2237	Calcarine_L	865	Precuneus_L	290
(with Top10 voxelsize >100)	Putamen_L	108	Insula_R	1557	Calcarine_R	546	Cuneus_R	282
			Frontal_Sup_L	1531	Cuneus_L	433	Occipital_Mid_L	258
			Rectus_R	822	Lingual_L	281	Occipital_Inf_L	100
			Rectus_L	601				
			Putamen_R	466				
			Hippocampus_R	453				
			OFCmed_R	323				
			OFCmed_L	315				
			ParaHippocampus_R	296				
			……					

The table demonstrates four clusters with significant differences in the RTT group compared with two control groups (FWE corrected, p < 0.05). Number of voxels and MNI coordinates of the peak voxel as well as the AAL labeling are listed. Age and total cranial volume were included as covariates.

### DTI-TBSS analyses

At the global level, the RTT group showed significantly decreased FA and increased MD in comparison with ASD and TD controls (Table 1 and Figure 2).

TBSS analyses were conducted between RTT group (*n* = 22), ASD controls (*n* = 23), and TD controls (*n* = 23). The whole brain TBSS revealed global reductions of FA in ANCOVA. In the *post-hoc* analysis, the RTT group showed extensively decreased FA compared to TD controls mainly in the corpus callosum, superior longitudinal fasciculus, anterior corona radiate, and some bilateralized tracts (*p* = 0.002, voxel size > 100; Table 3 and Figure 3). The comparison between RTT and ASD controls also showed significantly decreased FA mainly in the similar regions of tracts as in the RTT-TD comparison. For MD, the RTT group showed increased MD values compared to TD controls mainly in the body and genu of the corpus callosum (*p* = 0.046, voxel size > 100). When compared with ASD controls, the RTT group showed increased MD values mainly in the body, genu, and splenium of the corpus callosum, cingulum, anterior, and posterior corona radiata (Table 3 and Figure 3). No significant difference was found between the ASD and TD group comparisons with either an FA or MD value.

**Table 3:** Decreased FA and increased MD values in comparisons between the RTT group and controls for TBSS analysis.

			**Peak**			**FA (RTT < TD)**		**MD (RTT > TD)**	
**FA/MD**	**Voxels**	***P***	**MAX X (vox)**	**MAX Y (vox)**	**MAX Z (vox)**	**White matter tracts**	**Voxels (**>**100)**	**White matter tracts**	**Voxels**
**RTT vs. TD**									
FA	35,281	0.002	49	124	34	Body of corpus callosum	2919.64	Body of corpus callosum	738.53
MD	895	0.046	92	143	87	Genu of corpus callosum	1440.02	Genu of corpus callosum	156.39
						Splenium of corpus callosum	1352.98		
						Superior longitudinal fasciculus R	917.83		
						Anterior corona radiata R	609.23		
						Sagittal stratum R	245.27		
						Cingulum L	245.27		
						Posterior thalamic radiation L	237.37		
						Superior corona radiata L	229.46		
						Retrolenticular part of internal capsule R	197.8		
						Retrolenticular part of internal capsule L	197.8		
						Cingulum R	189.88		
						Anterior limb of internal capsule R	166.17		
						Posterior thalamic radiation R	158.23		
						Posterior corona radiata L	150.33		
						Sagittal stratum L	142.43		
						Cerebral peduncle R	134.52		
						External capsule R	102.84		
						……			
**RTT vs. ASD**									
FA	40,906	0.002	54	118	40	Body of corpus callosum	3025.48	Body of corpus callosum	2626.84
MD	4,903	0.006	102	157	80	Genu of corpus callosum	1556.64	Genu of corpus callosum	859.51
						Splenium of corpus callosum	1293.22	Splenium of corpus callosum	598.76
						Superior longitudinal fasciculus R	1077.67	Cingulum L	241.43
						Anterior corona radiata R	678.52	Anterior corona radiata L	135.20
						Superior longitudinal fasciculus L	534.84	Posterior corona radiata R	122.57
						Anterior corona radiata L	311.34		
						Posterior corona radiata R	295.35		
						Posterior thalamic radiation L	279.41		
						Superior corona radiata L	263.42		
						Cingulum L	247.47		
						External capsule L	239.48		
						Cerebral peduncle L	231.49		
						Posterior corona radiata L	207.55		
						Cingulum R	199.55		
						Retrolenticular part of internal capsule R	183.61		
						Retrolenticular part of internal capsule L	175.62		
						Anterior limb of internal capsule L	151.67		
						External capsule R	143.68		
						Posterior limb of internal capsule R	119.74		
						Sagittal stratum	111.75		
						……			

The table demonstrates clusters with significant differences (FWE corrected, p < 0.05) with number of voxels, p-values, and MNI coordinates of the peak voxel of each positive tract in the RTT group and two control groups. Age and total cranial volume were included as covariates. TBSS, tract-based spatial statistics.

### Correlations between clinical characteristics and brain morphology

In the RTT group, correlation results showed that the developmental quotient of the GDS-C social domain was related to the gray matter volumes of the left hippocampus, calcarine, rectus, and Rolandic operculum, as well as the right precuneus and rectus after the multiple corrections (Table 4). The developmental quotient of the GDS-C motor domain was related to the general FA value of TBSS. Moreover, the RSSS clinical severity score was correlated with the gray matter volumes of right putamen and the general FA value of TBSS after the multiple corrections (Table 4). However, no significant correlations were found in the ASD group.

**Table 4:** Correlations between clinical data and altered gray and white matter parameters in the RTT group.

**Clinical values brain parameters**	**Griffith_Social**		**RSSS**	
	**Coefficient**	***P*-value (2-tailed)**	**Coefficient**	***P*-value (2-tailed)**
GMV	0.411	0.041	−0.403	0.046
WMV	–	–	−0.408	0.045
TBSS_FA	0.446	0.038	−0.601*	0.003
TBSS_MD	–	–	0.422	0.037
Hippocampus_L	0.444*	0.026	−0.399	0.048
Hippocampus_R	0.410	0.041	−0.372	0.047
Precuneus_R	0.472*	0.017	−0.377	0.043
Cuneus_R	0.402	0.047	−0.433	0.031
Cuneus_L	0.411	0.041	–	–
Putamen_R	–	–	−0.500*	0.011
Precuneus_L	0.412	0.041	–	–
Frontal_Mid_L	–	–	−0.410	0.033
Calcarine_L	0.446	0.026	–	–
Calcatine_R	0.411	0.041	–	–
Lingual_L	0.428	0.037	–	–
Lingual_R	0.340	0.048	–	–
Rectus_L	0.449*	0.025	–	–
Rectus_R	0.452*	0.023	–	–
Rolandic_Oper_L	0.488*	0.013	–	–
	**Griffith_Motor**		**Griffith_Total**	
	**Coefficient**	***P*** **-value (2-tailed)**	**Coefficient**	***P*** **-value (2-tailed)**
TBSS_FA	0.468	0.002	0.422	0.034

The table demonstrates the correlation analysis between clinical data (GDS-C and RSSS) with gray and white matter parameters. Two-tailed p < 0.05 was set for a significant level. –, no significant correlation. *Survived with multiple corrections with a significant level at p < 0.05.

## Discussions

Our study demonstrated both gray and white matter morphometric features in the young RTT girls compared with age-paired ASD and TD controls. Pediatric-friendly pipelines were applied by generating study-specific tissue probability maps and templates during the preprocessing procedure. VBM analyses revealed decreased gray matter volumes mainly in the insula, frontal cortex, calcarine, and limbic/paralimbic regions. TBSS demonstrated decreased FA and increased MD mainly in the corpus callosum and other projection and association fibers such as superior longitudinal fasciculus and corona radiata. Furthermore, RTT social impairment and clinical severity were associated with these morphometric alterations. This study provides a unique opportunity to understand the underlying neuropathological mechanisms of RTT.

Since the first described as “progressive syndrome of autism” in 1983, RTT has been considered as one of the most severe subtypes of ASD for decades (Hagberg et al., 1983; Neul, 2012). However, it has been separated in the updated *DSM-5* (*Diagnostic and Statistical Manual of Mental Disorders, 5th edition*) as an independent neurologic disorder (Battle, 2013). Clinicians and specialists claimed that there were essential differences between RTT and ASD such as sex preference, eye contact, and issues with movements (Neul, 2012). However, most of the evidence was based on behaviors. Interestingly, this study exhibited a significant difference between RTT and both ASD and TD groups, but no significant difference between ASD and TD groups in both gray and white matter comparisons. These results may suggest different neuropathological mechanisms underlying RTT and ASD, thus providing more robust evidence to support the classification of RTT as an independent neurologic disorder from ASD.

Unlike idiopathic ASD, which has not been identified with a specific gene or set of genes, RTT is more of a monogenic disorder caused by *MECP2* gene variants (Neul et al., 2008, 2010). At the cellular level, the *MECP2* gene has been tested to express widely throughout the mature neurons of the brain and plays a crucial role in synaptic plasticity, neuronal development, and differentiation (Chahrour et al., 2008; Lyst and Bird, 2015; Kong et al., 2022). Autopsy studies in patients with RTT revealed a reduction in brain weight and an overall decreased brain size (Armstrong et al., 1995; Bauman et al., 1995; Armstrong, 2005). Early MRI studies confirmed the findings from the autopsy that RTT patients had global brain atrophy (Reiss et al., 1993; Subramaniam et al., 1997). More recent MRI studies using voxel-based methods have provided evidence of selective alterations in related brain regions such as cerebellum, occipital cortex, and anterior frontal lobe for the gray matter, as well as the corpus callosum and fibers in the frontal area for the white matter (Carter et al., 2008; Mahmood et al., 2010; Shiohama et al., 2019). MRI studies on *Mecp2*-null(KO) mice showed overlapping brain areas with decreased volume, supporting the hypothesis of regional brain reduction in RTT (Saywell et al., 2006; Akaba et al., 2022). In accordance with the previous studies, our results also exhibited more regional reductions in gray matter volume and more regional alterations in white matter parameters in girls with RTT.

To further compare with the previous voxel-based MRI studies in RTT patients, some gray matter volume decreased in regions overlapping with our findings, such as the frontal cortex and occipital cortex. However, the top volume reduced regions in our results also involved bilateral insula, which has not been reported in previous voxel-based studies in RTT patients. The possible reason could be that the ages of our participants are much younger (mean age of 3.5 ± 1.25 years); however, the previous voxel-based studies are either with older participants (mean age of 8.6–8.9 years; Carter et al., 2008) or with large life span (4.4–17.6 years; Shiohama et al., 2019). RTT patients usually progress through 4 clinical stages: Stage I is the early onset stage, with an onset age of 6–18 months; Stage II is the rapid destructive stage, with an onset age of 1–4 years; Stage III is the pseudo-stationary stage, with an onset age of 4–8 years; and Stage IV is the late deterioration stage, usually after 8 years (Hagberg and Witt-Engerstrom, 1986; Neul et al., 2010). The participants in previous voxel-based studies were mostly at Stages III–IV. It is worthy to mention that gross motor dysfunction, gait apraxia, stabilization issue, and truncal ataxia were usually represented by Stage III (Hagberg and Witt-Engerstrom, 1986; Neul et al., 2010). Thus, it is understandable why the previous voxel-based studies reported the cerebellum as the main affected region, which plays an important role in motor control. However, the subjects in this study were mostly in Stage II, and the results showed significant correlations between the brain morphometric features and Griffith social domain instead of the gross motor or fine motor domain. Furthermore, one study with younger RTT girls (mean age of 6 ± 2 years) applying magnetic resonance spectroscopy revealed lower NAA concentration in the insular cortex (Horska et al., 2000), which is consistent with this study. The insula is anatomically folded deep within the lateral sulcus receiving projection fibers from the thalamus, amygdala, frontal, and occipital cortex (Uddin et al., 2017; Rachidi et al., 2021). As one of the least understood brain regions, recent evidence suggests that the insula may play a role in certain higher-level functions such as socio-emotional, cognitive, and sensorimotor integration (Uddin et al., 2017). The consistency of our results between studies with younger RTT girls and the inconsistency between studies with older subjects may suggest underlying mechanisms for early-stage RTT pathogenesis. Unfortunately, similar to most patient studies, the majority of the Mecp2 animal models are with adult mice, and the early stages of development have not been well-characterized. Thus, future longitudinal studies in both human and animal models covering early age stages may allow for a more in-depth understanding of disease pathogenesis and progression.

Evidence suggested that the FA and MD values were positively related to the maturation of myelin and axon (Pecheva et al., 2018). Previous diffusion-weighted MRI studies reported reduced FA in tracts such as the corpus callosum, cingulum, and external capsule (Mahmood et al., 2010; Oishi et al., 2013). However, these studies used ROI-orientated fiber tracking instead of whole brain evaluation. In the current study, we applied TBSS, which is a fully automated approach in a voxel-wise manner for whole-brain analysis beyond *a priori*–defined tracts (Papma et al., 2017). Tracts with altered FA/MD values in this study were not only consistent with previously reported fibers such as corpus callosum, cingulum, and external capsule but also included other projection and association fibers, which indicated more comprehensive white matter alterations. Our current findings with comprehensive white matter alterations might provide *in vivo* evidence for the findings from studies on cellular levels, which propose that the MECP2 gene is expressed in nearly all the neurons and glia in the brain (Chahrour and Zoghbi, 2007; Qiu, 2018).

As a non-invasive imaging tool to obtain both structural and functional information about the *in vivo* brain, MRI has been widely introduced to identify specific pathological changes in the brain for neurodevelopmental disorders (Eliez and Reiss, 2000). Processing pediatric neuroimaging data is always a great challenge due to pervasive morphological changes during brain development. Considering the myelination maturation phase and brain tissue imaging contrast for MRI scans from children older than 2 years, we recommend processing study- or child-specific tissue probability maps or templates as an alternate to the standard adult templates which are embedded in the classical pipelines. To create the study-specific template for TBSS registration, we suggest simply changing the command line option to identify the “most representative” one as the target image instead of the adult FMRIB58_FA template image (https://fsl.fmrib.ox.ac.uk/fsl/fslwiki/TBSS). To create the study- or child-specific tissue probability maps for VBM preprocessing, we suggest the Template-O-Matic toolbox, which uses a large, healthy, reference database (*n* = 404) of children to generate matched templates for each subject based on age and gender (Wilke et al., 2008). Besides, this toolbox is quite user-friendly since the algorithm can be simply implemented in the form of the SPM toolbox. This procedure yields high-quality tissue maps to better match the individual input sample, which is a significant improvement over classical approaches, especially regarding spatial normalization and tissue segmentation processes (Wilke et al., 2008; Kozlowska et al., 2017).

There were some limitations to be considered. First, the current MRI scans were mainly structural, so future studies of functional connectivity may provide more in-depth evidence for our conclusions. Second, this study is cross-sectional. However, RTT is a postnatal neurodevelopmental disorder with symptoms getting worse with age. Thus, future longitudinal studies in both human and animal models may allow a better understanding of disease pathogenesis and progression.

## Conclusion

This study applied VBM for voxel-wised volumetric characteristics of gray matter and TBSS for white matter analysis in young girls with RTT using pediatric-adjusted analytic pipelines. Decreased gray matter in the insula, frontal cortex, and limbic/paralimbic regions as well as altered FA and MD values in the corpus callosum and other projection and association fibers provided some new guidance to further understand the underlying mechanisms in RTT.

## Data availability statement

The original contributions presented in the study are included in the article/supplementary material, further inquiries can be directed to the corresponding author/s.

## Ethics statement

The studies involving human participants were reviewed and approved by the Ethics Committee of Children's Hospital of Fudan University. Written informed consent to participate in this study was provided by the participants' legal guardian/next of kin.

## Author contributions

DL and LM analyzed and interpreted data and wrote and reviewed the manuscript. QX designed the research, analyzed and interpreted the data, and reviewed the manuscript. XX interpreted the data and reviewed the manuscript. ZQ interpreted the data and reviewed the manuscript. HL and CH collected the clinical data. BZ and KZ prepared the data and reviewed the manuscript. All authors contributed to the article and approved the submitted version.

## Funding

This study was supported in part by the National Natural Science Foundation of China (NSFC, No. 81701129, 82171540) and the Key Subject Construction Project of Shanghai Municipal Health Commission (No. shslczdzk02903).

## Conflict of interest

The authors declare that the study was conducted in the absence of any commercial or financial relationships that could be construed as a potential conflict of interest. The reviewer YY declared a shared parent affiliation with the authors to the handling editor at the time of review.

## Publisher's note

All claims expressed in this article are solely those of the authors and do not necessarily represent those of their affiliated organizations, or those of the publisher, the editors and the reviewers. Any product that may be evaluated in this article, or claim that may be made by its manufacturer, is not guaranteed or endorsed by the publisher.
